# Viral suppression among middle-aged and aging MSM living with HIV: Partnership type and quality

**DOI:** 10.1371/journal.pone.0258032

**Published:** 2021-10-01

**Authors:** Vaibhav Penukonda, Timothy Utz, Nicholas S. Perry, Deanna Ware, Mark Brennan-Ing, Steven Meanley, Andre Brown, Sabina Haberlen, James Egan, Steven Shoptaw, Linda A. Teplin, M. Reuel Friedman, Michael Plankey

**Affiliations:** 1 Georgetown University School of Medicine, Washington, District of Columbia, United States of America; 2 Department of Psychiatry, Rhode Island Hospital, Providence, Rhode Island, United States of America; 3 Center for Behavioral Medicine, The Miriam Hospital, Providence, Rhode Island, United States of America; 4 Department of Psychiatry and Human Behavior, Alpert Medical School of Brown University, Providence, Rhode Island, United States of America; 5 Department of Medicine, Division of Infectious Diseases, Georgetown University Medical Center, Washington, District of Columbia, United States of America; 6 Brookdale Center for Healthy Aging, Hunter College, City University of New York, New York, New York, United States of America; 7 Department of Family and Community Health, University of Pennsylvania School of Nursing, Philadelphia, Pennsylvania, United States of America; 8 Department of Behavioral and Community Health Sciences, Graduate School of Public Health, University of Pittsburgh, Pittsburgh, Pennsylvania, United States of America; 9 Department of Epidemiology, Bloomberg School of Public Health, Johns Hopkins University, Baltimore, Maryland, United States of America; 10 Department of Family Medicine, University of California, Los Angeles, California, United States of America; 11 Feinberg School of Medicine, Northwestern University, Chicago, Illinois, United States of America; 12 Department of Infectious Diseases and Microbiology, Graduate School of Public Health, University of Pittsburgh, Pittsburgh, Pennsylvania, United States of America; UNSW Australia, AUSTRALIA

## Abstract

Functional support—the availability of material aid, emotional support, or companionship—promotes general well-being. For men who have sex with men (MSM) living with HIV, having a person who supports you associates with viral suppression. This study examines the association between supportive partnerships and HIV viral suppression among middle-aged and aging MSM living with HIV. A total of 423 middle-aged and aging MSM (mean age, 58.2 years) from the Multicenter AIDS Cohort Study provided self-reported data about their partnerships. Separate Poisson regression models assessed how partnership type, support, strain, and duration from April 2017 were associated with repeated viral load measurements up to April 2019. Of the follow-up visits (N = 1289), 90.0% of participants were virally suppressed. Most participants reported being non-Hispanic White (61.0%) and college-educated (83.4%). Participants were asked about their primary partnerships (i.e., “someone they are committed to above anyone else”) and secondary partnerships (i.e., those who can also be intimate or supportive but not necessarily romantic or sexual). The participants reported: no partnerships (45.2%), only primary partnerships (31.0%), only secondary partnerships (11.1%), or both primary and secondary partnerships (12.8%). Primary and secondary partnerships had mean (SD) durations of 15.9 (11.3) and 25.2 (16.5) years, respectively. Participants reporting both primary and secondary partnerships (compared with no partnership) showed significantly higher odds of being virally suppressed (adjusted prevalence ratio [aPR], 1.04; 95% CI, 1.00–1.08; p = 0.043). Albeit not statistically significant, primary-only (aPR, 1.01; 95% CI, 0.97–1.06; p = 0.547) or secondary-only (aPR, 1.03; 95% CI, 0.98–1.08; p = 0.224) partnership types were positively associated with viral suppression. Partner support and strain were not associated with viral suppression in any partnership group. Being older and non-Hispanic Black were positively and negatively associated with viral suppression, respectively. Encouraging partnerships should be considered one of clinicians’ many tools to help middle-aged and aging MSM achieve long-term viral suppression.

## Introduction

Maintaining an undetectable viral load is essential to the health of men living with HIV and imperative in the treatment-as-prevention paradigm [1]. Viral suppression is achieving a viral load of fewer than 200 copies HIV RNA/mL [2–4]. In 2018, people aged 55 years and older represented the largest age group (32.3%) of all people living with HIV (PLHIV) in the U.S., with only 67% virally suppressed [5]. However, among Ryan White HIV/AIDS Program participants in 2018, men who have sex with men (MSM) comprised 56.4% of men living with HIV aged 50 years and older, and of this group, 93.6% had remained virally suppressed [6]. Despite this high suppression rate among MSM living with HIV who are enrolled in care, identifying factors that promote viral suppression in aging PLHIV is essential to the goal of reaching 90% viral suppression nationally by 2030 [7].

Research has consistently shown that functional support is positively associated with achieving viral suppression [8–10]. Functional support includes not only instrumental support in the forms of material aid and information, but also emotional support and companionship [11]. In a community sample of MSM, Friedman et al. [12] showed that having “someone you can count on for understanding or support” was positively associated with viral suppression. This is notable given that Statz et al. [13] found that 67.9% of middle-aged and aging (≥40 years) MSM had supportive partnerships; however, how supportive partnerships promote viral suppression in MSM living with HIV demands further exploration.

The impact of supportive partnerships on achieving viral suppression may be related to partnership type and quality. Partnerships may be categorized as primary or secondary, wherein primary partnerships are rooted in committed, romantic bonds, while secondary partnerships may be as intimate or supportive as primary partnerships but do not necessarily include romance or sex. The functional support that these partnerships provide may influence viral suppression [12]. Further, it might be assumed that primary partnerships would have a stronger influence on health outcomes based on the large literature on the association between romantic relationships and health [14].

Moreover, as posited by Slatcher and Selcuk’s strength and strain model [14], positive relationship qualities, such as functional support (e.g., material aid, emotional support, or companionship), are associated with better health outcomes, while negative relationship qualities, such as partner strain (e.g., demands, criticism), are associated with poor health outcomes. Among MSM primary partnerships, Johnson et al. [15] found that higher partner commitment, one measure of positive partnership quality, was positively associated with lower viral loads. Therefore, the supportive qualities of partnerships may be important in achieving undetectable viral load levels [16].

The current study aims to contextualize both Friedman and colleagues’ and Slatcher and Selcuk’s work in the context of the supportive partnerships of middle-aged and aging MSM living with HIV [12, 14]. Partnership type and the quality of said partnerships were evaluated for their associations with viral suppression among middle-aged and aging MSM in the Multicenter AIDS Cohort Study (MACS). Based on the work of Friedman et al and the model by Slatcher and Selcuk, we hypothesized that all partnership types provide functional support that is positively associated with viral suppression and that supportive and strained partnerships are associated with higher and lower odds of viral suppression, respectively.

## Materials and methods

### Study population

The MACS is a longitudinal cohort of HIV-positive and HIV-negative MSM at four sites in the United States: Baltimore, Maryland/Washington, DC; Chicago, Illinois; Los Angeles, California; and Pittsburgh, Pennsylvania/Columbus, Ohio. More than 7,000 participants have been enrolled in the study since 1984. Participants attended semiannual clinic visits in which medical history information and biomedical specimens were collected using an Audio Computer-Assisted Self-Interview and a standardized clinical examination. Details on the MACS study design has been described elsewhere [17, 18]. The Understanding Patterns of Healthy Aging Among Men Who Have Sex With Men substudy of the MACS included approximately 1,300 participants across six semiannual visits (2016 to 2019). Eligibility criteria were (1) being at least 40 years of age at the substudy’s commencement; (2) completing a MACS visit in the two years prior to substudy enrollment; and (3) reporting at least one sexual encounter with another man since enrolling in the MACS [19]. The analytic sample included 423 HIV-positive participants who responded to questions related to primary and secondary partnerships during visit 67 (April 2017–September 2017). The data were fully anonymized before we accessed them. The institutional review boards at John Hopkins University; Northwestern University; University of California, Los Angeles; and University of Pittsburgh approved the protocol, and written informed consent was obtained from all study participants.

### Outcome

Viral load suppression (suppressed/not suppressed) was assessed at visits 68 (October 2017–March 2018), 69 (April 2018–September 2018), and 70 (October 2018–March 2019). It was defined as having plasma HIV RNA levels (viral load) of less than or equal to 200 copies per mL [2–4].

### Primary predictors

Partnership status was ascertained from self-reported primary and secondary partner questions. Primary partner status was obtained from the following question: “Are you currently in a relationship with a primary partner? By primary partner we mean someone who you are committed to above anyone else and with whom you might or might not be having sex.”

Participants who answered affirmatively to this were categorized as having a primary partner. Secondary partner status was obtained from the following question: “We know that some gay and bisexual men form partnerships with other people that can be as intimate or supportive as a primary partnership or a spouse, but that don’t necessarily include romance or sex. Similar to a primary partner or spouse, this individual might be someone who shares financial resources to pay living expenses, shares housing, shares personal sacred histories between both of you, or takes cares of you when seriously ill (or you them). Do you have someone like that in your life currently?” Participants who answered affirmatively to this were categorized as having a secondary partner. Secondary partnerships are characterized as one of the following types: (1) biological family; (2) chosen family; (3) polyamorous/additional romantic partner; (4) close friend; (5) former romantic partner; and (6) current or former sexual partner. Participants were allowed to select multiple descriptors regarding their secondary partnership. Next, partnership type was derived from reported primary and secondary partner statuses and categorized as follows: (1) no primary or secondary partners; (2) secondary partner and no primary partner; (3) primary partner and no secondary partner; and (4) both primary and secondary partners. Partnership status was assessed at visit 67.

Partnership support and strain were measured as continuous variables using a scale from the Midlife in the United States survey [20]. Each subscale was composed of four items that were adapted so that they read, “thinking about your primary partner.” The four items from the support subscale were: “How much does your partner understand the way you feel about things?”; “How much does your partner really care about you?”; “How much can you rely on your partner for help if you have a serious problem?”; and “How much can you open up to your partner if you need to talk about your worries?” The four items from the strain subscale were: “How often does your partner criticize you?”; “How often does your partner make too many demands on you?”; “How often does your partner let you down when you are counting on him/her?”; and “How often does your partner get on your nerves?” Participants rated each item on a 5-point Likert scale, ranging from 1, indicating “never,” to 5, indicating “regularly.” Scores for each subscale were summed. The scores for both the support and strain subscales ranged from 4 to 20, with higher values indicating higher partnership support or strain. Participants who reported secondary partnerships were asked similar questions about their secondary partners. The standardized Cronbach alphas for primary partnership support and strain were 0.89 and 0.78, respectively, and for secondary partner support and strain were 0.89 and 0.84, respectively, in our sample. In the model, estimates were presented as 3-unit increases in support and strain scores, which represented a single standard deviation increase in this sample. Support and strain were assessed at visit 67.

Participants who reported a primary or secondary partnership were asked about the duration of their primary or secondary partnership in years. Partnership duration was assessed at visit 67.

### Covariates

Participants’ chronological age at visit 67 was calculated from self-reported date of birth and date of visit. Race/ethnicity and education were obtained on enrollment into the MACS. Race/ethnicity was categorized as non-Hispanic White, non-Hispanic Black, Hispanic, and other. Education was categorized as less than a high school diploma, high school diploma, college, and graduate school. For modeling purposes, education was collapsed into “less than college” and “some college or higher.” Enrollment wave refers to the time in which participants enrolled in the MACS and was categorized as original cohort, 2001–2003 cohort, and 2011–2019 cohort.

### Statistical analyses

Descriptive statistics on the outcome were generated, including primary predictors and covariates by viral load category (suppressed vs not suppressed). Separate Poisson regression models with robust error variance were used to test the associations of the following primary predictors and covariates at visit 67 on repeated viral load suppression outcomes at visits 68, 69, and 70 [21]: (1) partnership type (primary, secondary, both, or none); (2) primary partner support/strain and partnership duration among participants reporting primary partnerships; (3) secondary partner support/strain and partnership duration among participants reporting secondary partnerships; and (4) primary and secondary support/strain among participants reporting both primary and secondary partnerships. For each model, the primary predictors and covariates were independently tested. We included covariates with a p-value less than or equal to 0.1 and the primary predictors in the adjusted models. The model dropped any observation missing viral load across all three time points. In this analysis, there were only 4 missing assessments out of 423 viral load measurements (<1%) across all three time points. While these data were excluded from analysis, they are included in the descriptives in Table 1. Data were analyzed using SAS version 9.4 (SAS Institute Inc). We reported unadjusted and adjusted prevalence ratios (PR) and 95% CIs.

**Table 1:** Population characteristics by partnership type.

	No Primary or Secondary Partnerships	Primary-Only Partnership	Secondary-Only Partnership	Both Primary and Secondary Partnerships	Total
**N (%)**	191 (45.2%)	131 (31.0%)	47 (11.1%)	54 (12.8%)	423 (100%)
**Age, mean (SD) [minimum-maximum], years**	58.4 (8.2) [41–78]	57.4 (7.6) [41–71]	60.0 (7.2) [41–74]	58.0 (7.4) [41–74]	58.2 (7.8) [41–78]
**Race/ethnicity, n (%)**					
** Non-Hispanic White**	107 (56.0%)	91 (69.5%)	29 (61.7%)	31 (57.1%)	258 (61%)
** Non-Hispanic Black**	58 (30.4%)	25 (19.1%)	14 (29.8%)	15 (27.8%)	112 (26.5%)
** Hispanic**	22 (11.5%)	12 (9.2%)	4 (8.5%)	7 (13.0%)	45 (10.6%)
** Other**	4 (2.1%)	3 (2.3%)	0 (0.0%)	1 (1.9%)	8 (1.9%)
**Education, n (%)**					
** Less than high school diploma**	11 (5.8%)	4 (3.1%)	2 (4.3%)	0 (0.0%)	17 (4.0%)
** High school diploma**	24 (12.6%)	15 (11.5%)	6 (12.8%)	8 (14.8%)	53 (12.5%)
** At least some college work**	102 (53.4%)	70 (53.4%)	25 (53.2%)	32 (59.3%)	229 (54.1%)
** At least some graduate work**	54 (28.3%)	42 (32.1%)	14 (29.8%)	14 (25.9%)	124 (29.3%)
**Enrollment wave, n (%)**					
** Original cohort**	97 (50.8%)	65 (49.6%)	32 (68.1%)	31 (57.4%)	225 (53.2%)
** 2001–2003 cohort**	71 (37.2%)	54 (41.2%)	13 (27.7%)	14 (25.9%)	152 (35.9%)
** 2011–2019 cohort**	23 (12.0%)	12 (9.2%)	2 (4.3%)	9 (16.7%)	46 (10.9%)
**Primary partner quality, mean (SD)**					
** Support**	-	15.9 (3.1)	-	15.8 (3.1)	15.9 (3.1)
** Strain**	-	8.7 (2.5)	-	9.1 (3.5)	8.8 (2.8)
**Secondary partner quality, mean (SD)**					
** Support**	-	-	15.2 (2.4)	15.4 (3.5)	15.3 (3.0)
** Strain**	-	-	8.5 (2.8)	7.7 (2.8)	8.1 (2.8)
**Duration of partnership, mean (SD), years**					
** Primary partner**	-	16.1 (10.7)	-	15.5 (12.6)	15.9 (11.3)
** Secondary Partner**	-	-	25.5 (17.7)	24.9 (15.6)	25.2 (16.5)
**Viral Load Suppression at visits 68, 69, and 70** ^**a**^					
** <200 copies/mL**	503 (88.3%)	365 (91.5%)	138 (92.6%)	154 (90.1%)	1160 (90.0%)
** ≥200 copies/mL**	36 (6.3%)	18 (4.5%)	3 (2.0%)	6 (3.5%)	63 (4.7%)
** Missing data**	31 (5.4%)	16 (4.0%)	8 (5.4%)	11 (6.4%)	66 (5.1%)

^a^n = 1289.

## Results

### Population characteristics

Overall, most participants (N = 423) were non-Hispanic White (61.0%), completed at least some college (83.4%), and were virally suppressed across visits 68, 69, and 70 (n = 1289; 90.0%). The mean (SD) age of the analytic sample was 58.2 (7.8) years. The distribution of enrollment wave was 53.2% from the original cohort, 35.9% from the 2001–2003 cohort, and 10.9% from the 2011–2019 cohort. The participants reported the following partnership statuses: (1) no primary or secondary partnerships (n = 191; 45.2%); (2) primary-only partnership (n = 131; 31.0%); (3) secondary-only partnership (n = 47; 11.1%); and (4) both primary and secondary partnerships (n = 54; 12.8%). The mean (SD) durations of primary and secondary partnerships were 15.9 (11.3) years and 25.2 (16.5) years, respectively. Among participants reporting primary partnerships, the mean support and strain scores ranged from 4 to 20 and had a mean (SD) value of 15.9 (3.1) and 8.7 (2.5), respectively. Among participants reporting secondary partnerships, the mean support and strain scores ranged from 4 to 20 and had a mean (SD) value of 15.2 (2.4) and 8.5 (2.8), respectively (Table 1). Higher scores indicated higher support or strain. We report sample characteristics by partnership type in Table 1.

### Association of partnership type, support and strain, and viral suppression

We reported unadjusted associations of partnership type, primary partnership support/strain, secondary partnership support/strain, partnership duration, covariates, and viral suppression in Table 2. Education was not included into the final multivariable models because the p-value in the unadjusted analysis was greater than 0.1. After adjusting for age and race/ethnicity, both primary and secondary partnerships were associated with a 4% higher prevalence of viral load suppression (aPR, 1.04; 95% CI, 1.00–1.08; p = 0.043) compared with those reporting no partnership (Table 3). Albeit not statistically significant, primary-only (aPR, 1.01; 95% CI, 0.97–1.06; p = 0.547) or secondary-only (aPR, 1.03; 95% CI, 0.98–1.08; p = 0.224) partnership types were also positively associated with viral suppression. Among participants reporting primary partnerships, we found that neither primary partner support and strain nor partnership duration was associated with viral load suppression (Table 4). Similarly, among participants reporting secondary partnerships, neither secondary partner support, strain, nor partnership duration was associated with viral load suppression (Table 5). Among participants reporting both primary and secondary partnerships, the effect of including both primary partner support/strain and secondary partner support/strain was not associated with viral load suppression (Table 6). In the adjusted partnership type model (Table 3), a five-year increase in age was associated with higher prevalence of viral suppression (aPR, 1.01; 95% CI, 1.00–1.03; p = 0.032). Participants who reported being non-Hispanic Black (aPR, 0.92; 95% CI, 0.88–0.97; p = 0.003) had lower prevalence of viral load suppression compared with participants who reported being non-Hispanic White. We reported the association of age and race/ethnicity with viral load suppression in the other adjusted models in detail in Tables 4–6.

**Table 2:** Unadjusted association of partnership type, primary partnership quality, secondary partnership quality, covariates, and viral load suppression.

	Unadjusted Prevalence Ratio (95% CI)	p-value
**Partnership type**		
** Primary-only partnership**	1.02 (0.98–1.06)	0.329
** Secondary-only partnership**	1.03 (0.98–1.08)	0.230
** Both primary and secondary partnerships**	1.05 (1.01–1.09)	0.011
** No primary or secondary partnerships (referent)**		
**Primary partnership quality (per 3-unit increase)**		
** Support**	1.00 (0.98–1.02)	0.948
** Strain**	1.00 (0.98–1.02)	0.863
**Secondary partnership quality (per 3-unit increase)**		
** Support**	1.01 (0.98–1.03)	0.680
** Strain**	0.99 (0.97–1.01)	0.428
**Age (per 5-year increase)**	1.02 (1.01–1.03)	0.001
**Race/ethnicity**		
** Non-Hispanic Black**	0.91 (0.87–0.95)	<0.001
** Hispanic**	0.90 (0.82–0.97)	<0.001
** Other**	0.99 (0.93–1.05)	0.734
** Non-Hispanic White (referent)**	-	-
**Education**		
** Some college or higher**	1.02 (0.98–1.06)	0.355
** Less than college (referent)**	-	-
**Enrollment wave**		
** 2001–2003 cohort**	0.95 (0.92–0.99)	0.009
** 2011–2019 cohort**	0.93 (0.87–1.00)	0.047
** Original cohort (referent)**	-	-
**Duration of partnership (per 5-year increase)**		
** Primary partner**	1.01 (1.00–1.02)	0.020
** Secondary partner**	1.01 (1.00–1.01)	0.053

**Table 3:** Adjusted association of partnership type and viral load suppression.

	Adjusted Prevalence Ratio (95% CI)	p-value
**Partnership type**		
** Primary-only partnership**	1.01 (0.97–1.06)	0.547
** Secondary-only partnership**	1.03 (0.98–1.08)	0.224
** Both primary and secondary partnerships**	1.04 (1.00–1.08)	0.043
** No primary or secondary partnerships (referent)**	-	-
**Age (per 5-year increase)**	1.01 (1.00–1.03)	0.032
**Race/ethnicity**		
** Non-Hispanic Black**	0.92 (0.88–0.97)	0.003
** Hispanic**	0.95 (0.88–1.02)	0.137
** Other**	1.01 (0.92–1.10)	0.849
** Non-Hispanic White (referent)**	-	-
**Enrollment wave**		
** 2001–2003 cohort**	1.02 (0.97–1.06)	0.463
** 2011–2019 cohort**	0.99 (0.92–1.07)	0.808
** Original cohort (referent)**	-	-

**Table 4:** Adjusted association of partnership quality and viral load suppression among participants reporting primary partnerships.

	**Adjusted Prevalence Ratio (95% CI)**	**p-value**
**Primary partnership quality (per 3-unit increase)**		
** Support**	1.00 (0.97–1.02)	0.738
** Strain**	1.00 (0.98–1.03)	0.811
**Age (per 5-year increase)**	1.02 (1.00–1.04)	0.095
**Race/ethnicity**		
** Non-Hispanic Black**	0.91 (0.83–0.99)	0.038
** Hispanic**	0.95 (0.85–1.06)	0.345
** Other**	0.97 (0.81–1.16)	0.704
** Non-Hispanic White (referent)**	-	-
**Primary partnership duration (per 5-year increase)**	1.01 (1.00–1.02)	0.135
**Enrollment wave**		
** 2001–2003 cohort**	1.01 (0.96–1.06)	0.791
** 2011–2019 cohort**	0.94 (0.82–1.07)	0.320
** Original cohort (referent)**	-	-

**Table 5:** Adjusted association of secondary partnership quality and viral load suppression among participants reporting secondary partners.

	Adjusted Prevalence Ratio (95% CI)	p-value
**Secondary partnership quality (per 3-unit increase)**		
** Support**	1.01 (0.98–1.04)	0.749
** Strain**	0.99 (0.96–1.01)	0.342
**Age (per 5-year increase)**	1.00 (0.98–1.01)	0.579
**Race/ethnicity**		
** Non-Hispanic Black**	0.93 (0.86–1.01)	0.051
** Hispanic**	0.91 (0.78–1.07)	0.241
** Other**	0.94 (0.87–1.12)	0.148
** Non-Hispanic White (referent)**	-	-
**Secondary partnership duration (per 5-year increase)**	1.00 (1.00–1.01)	0.212
**Enrollment wave**		
** 2001–2003 cohort**	1.05 (0.98–1.12)	0.150
** 2011–2019 cohort**	1.07 (1.00–1.13)	0.036
** Original cohort (referent)**	-	-

**Table 6:** Adjusted association of partnership and secondary partnership quality and viral load suppression among participants reporting both primary and secondary partnerships.

	Adjusted Prevalence Ratio (95% CI)	p-value
**Primary partnership quality (per 3-unit increase)**		
** Support**	0.99 (0.95–1.03)	0.669
** Strain**	0.99 (0.95–1.02)	0.444
**Secondary partnership quality (per 3-unit increase)**		
** Support**	1.01 (0.97–1.05)	0.552
** Strain**	0.98 (0.94–1.02)	0.312
**Age (per 5-year increase)**	0.98 (0.96–1.01)	0.206
**Race/ethnicity**		
** Non-Hispanic Black**	0.91 (0.82–1.02)	0.118
** Hispanic**	0.90 (0.82–1.02)	0.309
** Other**	0.96 (0.82–1.12)	0.486
** Non-Hispanic White (referent)**	-	-
**Duration of partnership (per 5-year increase)**		
** Primary partner**	1.01 (1.00–1.02)	0.224
** Secondary partner**	1.01 (0.99–1.02)	0.334
**Enrollment wave**		
** 2001–2003 cohort**	1.04 (0.93–1.17)	0.486
** 2011–2019 cohort**	1.06 (0.97–1.15)	0.178
** Original cohort (referent)**	-	-

## Discussion

### Primary findings

Compared to middle-aged and aging MSM living with HIV without a primary or secondary partner, we found that having multiple sources of support, specifically both a primary and secondary partner, was positively associated with viral load suppression. Additionally, reporting only a primary or only a secondary partnership was also positively associated with viral load suppression, although not statistically significant. Our findings support other published evidence of a positive association between functional support and viral load among PLHIV [8–10]. Friedman et al. [12] found that increased levels of functional support—for example, having someone you can count on for understanding or support—was positively associated with viral suppression among MSM living with HIV. Other research has shown that, among PLHIV, functional support from a partner showed greater association with antiretroviral therapy (ART) adherence than that from friends and family [10, 22]. Support from partnerships may help compensate for the various risk factors that can preclude one from viral suppression. Specifically, supportive partners may provide a stress-buffering effect, thereby reducing the potential for stress to decrease medication adherence and, in turn, lead to poorer viral control.

In terms of partnership quality, we found that increased partner support and strain did not alter the odds of viral load suppression. We expected but did not find that partnership quality (i.e., high support, low strain) explained how partnerships promote viral suppression based on Slatcher and Selcuk’s model [14]. Under this model, one would have expected support from partnerships to have a stress-buffering effect on outside stressors (e.g., chronic disease, financial pressure, work) leading to higher rates of viral suppression, while partnership strain would have exacerbated outside stressors and thus, potentially, led to lower rates of viral suppression [14]. Indeed, our finding was unexpected given the prevailing understanding that supportive relationships positively impact health among the general population [23–25]. Previous work by Johnson et al. [15] among MSM living with HIV in serodiscordant and seroconcordant partnerships tested the association between viral suppression and feelings of commitment, intimacy, autonomy, and equality among both individuals in a primary partnership. Higher commitment was positively associated with lower viral load; however, no other measures of quality were associated with viral suppression, despite fewer participants with an undetectable viral load (76.9%) compared with that of our study (90.0%) [15]. The results of our study and that of Johnson et al. might suggest that being in a partnership is associated with better viral suppression, more so than the quality of said partnership. However, given that our finding is contrary to prior theory, further exploration of why this might be the case is certainly warranted.

### Secondary findings

Older age was positively associated with higher odds of viral suppression. Our finding supports prior studies showing that adherence to medications increases with age [26]. Ghidei et al. [27] showed that individuals older than 50 years taking ART have a reduced risk of nonadherence when compared with their younger counterparts. These older individuals have a higher tolerance to ART, as they cite fewer reasons to switch treatment, such as nausea or need for a simpler regimen [28]. Furthermore, among HIV-positive LGBT adults, aging may promote self-acceptance and a will to live that enhances adherence to medications [29, 30].

Non-Hispanic Black participants had lower odds of viral suppression compared with non-Hispanic White participants. The lower odds cannot be explained by differences across partnership status by race/ethnicity. Having limited access to ART, being unstably housed, or having a lower income have been shown to be associated with lower suppression among Black MSM [31]. It is important to consider how these factors are related to larger systemic social adversities. A study controlling for the disproportionately lower access to healthcare, greater perceived HIV stigma, racial discrimination, and multi-morbidities that Black MSM face could more accurately explain the relationship between social support and viral suppression among Black, non-Hispanic MSM [32, 33]. In addition to the lack of supportive partnerships, we postulate that these factors unmeasured in our study may explain differences in viral suppression by race/ethnicity [13, 31, 34].

Notable in our sample was the high proportion of middle-aged and aging MSM with no primary or secondary partnerships (45.2%). This finding is concerning because lower levels of perceived social support have been shown to be negatively associated with ART adherence among MSM living with HIV [35, 36]. The high proportion of older MSM without partners resembles existing data on social isolation among aging PLHIV. Emlet et al. [37] found that 39% of adults older than 50 years living with HIV were socially isolated compared with 25% of younger PLHIV. The lack of partnered MSM in our sample may also be explained by MSM seeking fewer, new partners as they age or the loss of former partners, both of which potentially contribute to reduced levels of perceived functional support. Underpinning an individual’s control over their social connections, the internalization of systemic homophobia, ageism, and HIV-related stigma may preclude aging HIV-positive MSM from seeking social support [38].

### Limitations

There are several limitations to our study. First, the sample was 90.0% virally suppressed and, therefore, the variance of undetectability was small. While we do not underestimate the community importance of a high prevalence of viral suppression in this sample, this high prevalence may explain the lack of a statistically significant association between viral suppression and participants with only primary or only secondary partnerships, as well as the nonsignificant associations with partnership support and strain. The level of detectable viral loads might have been higher if we had a larger sample size and or if more participants had completed the partnership questions: of the 41 participants (8.8%) who were left out of the current analyses due to missing partnership data, 17.1% had a detectable viral load compared with just 6.2% with a detectable viral load in the data analysis sample (participants who were not missing partnership data). However, there was no appreciable change in the pattern of results when missing data were accounted for. Second, the MACS is a convenient sample, and although it has a longitudinally well-characterized cohort of MSM, the results may not be generalizable to the larger population of MSM living with HIV where there may be more variability in viral load. Additionally, our sample was predominantly White, therefore our ability to analyze racial differences was limited. Third, data on partnerships were self-reported, an unavoidable limitation of survey studies. Despite these considerations, this work expands the understanding that social support is associated with better viral suppression among older MSM living with HIV.

## Conclusions

Based on this study, clinicians involved in the management of middle-aged and aging MSM living with HIV should invite their patients to consider the question, “Who can you call on for support?” One use of this question is for clinicians to inform primary and secondary partners of the important role their support plays in their loved one’s long-term adherence and undetectability [39]. For patients who answer “No one” to the aforementioned question, clinicians should remember that functional support is an important part of their clinical toolkit and that they may encourage patients to seek functional support. A conversation about the benefits of supportive partnerships would particularly aid those patients who have a detectable viral load or who experience treatment fatigue [40]. Although many psychosocial factors govern the establishment and maintenance of close partnerships, clinicians should motivate patients to seek functional support within their community [41].
